# Pretense and Executive Function: Testing the Roles of Representational Hierarchical Complexity and Embodiment

**DOI:** 10.3390/bs16091495

**Published:** 2026-08-26

**Authors:** Ke Yin, Qiong Wu, Xiaosong Gai

**Affiliations:** 1 Faculty of Education, Northeast Normal University, Changchun 130024, China; yink996@nenu.edu.cn (K.Y.); wuq936@nenu.edu.cn (Q.W.); 2 School of Psychology, Northeast Normal University, Changchun 130024, China

**Keywords:** pretense, executive function, representation, embodiment, iterative reprocessing

## Abstract

Pretense has been linked to children’s executive function, but less is known about whether specific characteristics of pretense influence subsequent executive function performance. Drawing on the Iterative Reprocessing (IR) model and research on embodied cognition, this study examined the roles of representational hierarchical complexity and embodiment in children’s immediate executive function performance following a single story-based pretense activity. A 2 (representational hierarchical complexity: primary vs. secondary) × 2 (embodiment: embodied vs. disembodied) between-subjects pre-test–post-test design was used with 131 first-grade children (M = 6.90 years). After correction for multiple testing across executive function outcomes, no significant main effects or interactions were found for inhibition, working memory, shifting, or the executive function composite. In the unadjusted analysis, an interaction between representational hierarchical complexity and embodiment emerged for inhibitory control, but this effect did not survive multiplicity correction and should therefore be considered exploratory. Overall, the findings do not provide robust evidence that varying representational hierarchical complexity or embodiment within a single pretense session influences children’s subsequent executive function performance. The exploratory inhibition pattern warrants further testing in independent studies.

## 1. Introduction

Executive function (EF), also known as cognitive control, refers to a set of psychological processes that individuals use to focus attention, maintain goals in mind, and resist inappropriate automatic responses. These processes include three primary components: inhibition (inhibitory control), working memory, and shifting (Miyake et al., 2000; Diamond, 2013). EF develops rapidly during adolescence and earlier stages of life and significantly predicts both short-term outcomes, such as academic achievement, and long-term development, including physical health and wealth (Peng & Kievit, 2019; Morgan et al., 2019; Moffitt et al., 2011).

In executive function training research, computerized training has been an early and successful intervention (Diamond, 2013), but it has limitations. First, once the computerized training concludes, participants are unable to continue similar exercises in daily life, leading to difficulties in sustaining training benefits (Diamond & Ling, 2016). Second, computerized training requires prolonged sitting, which is detrimental to children’s physical health (Martinovic et al., 2015). Third, the far transfer effects of computerized training are not sufficiently evident (Kassai et al., 2019). Therefore, given the limitations of computerized training, using real-world activities for training may offer a more effective alternative (Diamond & Ling, 2016).

Pretense is one of the most favored activities in early childhood and centrally involves a form of representation or acting-as-if (Weisberg, 2015; Thompson & Goldstein, 2019). Pretense may play a significant role in the development of children’s executive function (Constien et al., 2026; Doebel & Lillard, 2023). This relationship can be understood from the following perspectives. Vygotsky (1978) emphasized that through pretense, children develop self-control by adhering to collective rules, coordinating their behaviors, and cooperating with others. Additionally, as they differentiate between the physical attributes of objects and their symbolic meanings, children gradually learn to act based on internal representations rather than external reality (Vygotsky, 1967, 1978), thereby enhancing their ability to regulate behavior effectively. Pretense may also engage creative processes reliant on executive function (Vasilopoulos & Dumontheil, 2024; Bunce & Woolley, 2021; Thibodeau-Nielsen et al., 2020a), as children generate alternative scenarios while inhibiting immediate reality and maintaining and updating representations of the fictive play environment.

Many empirical studies provide evidence that pretense can support the development of children’s executive functions. First, correlational evidence demonstrates links between pretense and EF. Children’s ability to understand others’ pretense has been found to be positively correlated with inhibition (Van Reet, 2020). Furthermore, the frequency/duration of engaging in pretense in preschool children has been consistently observed to associate with better concurrent EF performance (Metaferia et al., 2020a, 2020b; Metaferia et al., 2021) and has been demonstrated to positively predict longitudinal gains in EF after 1–2 years (Thibodeau-Nielsen & Gilpin, 2020; Thibodeau-Nielsen et al., 2020b; R. E. White et al., 2021). Notably, as pointed out by Lillard et al. (2013), correlational findings cannot establish the directionality of this relationship; they provide only a necessary precondition for a causal influence of pretense on EF. Second, experimental evidence provides more direct support for the role of pretense in the development of children’s executive functions. Multiple experimental studies have shown that children who engage in pretense activities perform better on executive function tasks. For example, children who acted out a story after listening to it outperformed those engaged in non-pretense activities (such as pasting story-related cutouts onto a storyboard) on an inhibition task (R. E. White & Carlson, 2021). Similarly, children who engaged in superhero pretense (i.e., an individual who continues to perform exceptionally well even when the task becomes challenging) during executive function tasks not only performed better on switching tasks than children who did not pretend or only used a first-person perspective (R. E. White & Carlson, 2016), but also exhibited significantly greater task persistence (R. E. White et al., 2017). Moreover, Thibodeau et al. (2016) further found that, after a five-week intervention, children assigned to a pretense condition (encouraged to engage in thematic/scenario-based pretense, e.g., a trip to the moon) showed significantly greater improvements in EF compared to both a non-pretense condition (involving action-oriented activities such as ball games and coloring) and a control condition (continuing regular classroom activities without pretense).

Building on prior research emphasizing the role of representation (Vygotsky, 1978; Thibodeau-Nielsen et al., 2020a), the Iterative Reprocessing (IR) model specifies how individuals enhance the hierarchical complexity of representations through reflection or reprocessing (Zelazo, 2015, 2020), underscoring the importance of representational complexity and offering a novel account of how pretense facilitates executive function development. According to this model, as children’s capacity for reflection becomes more efficient, they are increasingly capable of reprocessing information in an iterative manner, enabling them to construct more sophisticated and higher-order representations. These enriched representations, in turn, support the formation, maintenance, and flexible use of behavior-guiding rules within working memory. The increased complexity of these rules is proposed to directly support the development of executive function. For instance, Zelazo (2015) proposed that, in the absence of reflective processing, individuals tend to rely on pre-existing associations and form relatively simple representations of a stimulus. A child may therefore initially represent a plush bear merely as a “bedtime companion.” Through reflective reprocessing, however—for example, by considering “What if the bear gets sick?”—the child can construct a more complex and layered representation of the same stimulus, such that the bear may be treated as a patient when it is “sick” but as a bedtime companion when it is not. Such representational elaboration supports the use of more complex behavior-guiding rules and, in turn, engages executive function processes (Cunningham et al., 2007).

Within the IR framework, representational complexity primarily refers to representational hierarchical complexity, indexed by the degree of embedding in the rule systems that must be formed and maintained in working memory (Zelazo et al., 2003; Zelazo, 2015). Rules are construed as conditional structures linking antecedents and consequents (e.g., “If I see a plush bear [antecedent], then I should treat it as a bedtime companion [consequent]”). Through reflection, children can consider lower-order rules in relation to one another and subordinate them to higher-order rules that specify the conditions under which particular lower-order rules should be selected and applied. Representational hierarchical complexity therefore increases with the number of degrees of embedding in the resulting rule system. For example, a higher-order rule in pretense might specify, “If we are playing hospital, then if the bear is sick, take it to the doctor and give it medicine, but if the bear is not sick, let it rest; whereas if we are playing bedtime, then if the bear is tired, put it to bed, but if it is not tired, continue playing.” Here, the higher-order setting condition (e.g., whether the child is playing hospital or bedtime) governs which set of lower-order conditional rules should be selected and applied. Such rule embedding reflects an advancement in the structural complexity of the child’s cognitive representations. Importantly, classic theory of mind tasks, such as second-order false-belief reasoning (e.g., “She thinks that he thinks…”), require children to embed one mental state within another. Such higher-order mental-state reasoning similarly entails an additional level of representational embedding, increasing the hierarchical structure of the representations that must be maintained and coordinated (Frye et al., 1995; Zelazo et al., 2003). These examples illustrate how increasing levels of embedding can give rise to progressively more hierarchically complex representations. In pretense, variations in the hierarchical complexity of the representations children construct may therefore create different opportunities for reflection and reprocessing, with potential implications for executive function.

In addition to its representational complexity, pretense can be embodied to varying degrees. This embodiment arises from cognitive processes situated within a dynamic body–environment interaction system, shaped by body movements, posture, and environmental engagements (Foglia & Wilson, 2013; Glenberg, 1997; Glenberg et al., 2013; Stolz, 2015; Wilson, 2002). From research on embodiment and the Iterative Reprocessing model (Zelazo, 2015), two central insights emerge. First, greater representational hierarchical complexity affords more opportunities for children’s reflection and reprocessing (Zelazo, 2015). Second, the degree of embodiment alters the extent to which cognition is shaped by bodily engagement and environmental affordances. High-embodiment activities (e.g., wearing a doctor’s coat while manipulating a stethoscope and mimicking injections with real props) increase bodily engagement and perceptual experience, thereby strengthening body–environment integration, fostering deeper connections between activity content and children’s prior beliefs and knowledge, and enhancing children’s engagement in the activity itself (Fiorella & Mayer, 2016; Castro-Alonso et al., 2021, 2024). By contrast, low-embodiment activities (e.g., verbally stating “I am being a teacher” using mini props) provide limited bodily engagement and sensory feedback, resulting in weaker body–environment integration (Pouw et al., 2020; Zhang et al., 2023; Abrahamson et al., 2020). Based on these perspectives, this article identifies representational hierarchical complexity and embodiment as two features of pretense that may help explain variation in children’s executive function performance: (1) Pretense involving higher representational hierarchical complexity may increase play challenge, create more opportunities for reflection, demand greater reprocessing, and enhance reflection efficiency, thereby potentially supporting the construction, maintenance, and flexible application of behavior-guiding rules in working memory. (2) High-embodiment pretense (e.g., involving actions and real props) may promote executive function by engaging children’s bodies directly (e.g., coordinating speech and gestures with imagined scenarios or symbolic substitutions), which may enhance situational realism and strengthen psychological involvement. Stronger psychological involvement, in turn, may motivate greater self-reflection and reprocessing for regulating one’s behavior within the pretend scenario and adhering to its imagined rules. This involvement-driven self-regulation may further enhance reflection efficiency, supporting the construction, maintenance, and flexible application of behavior-guiding rules in working memory, and potentially facilitating executive function development. Examining these two features may help clarify whether and how representational and embodied demands jointly shape executive function performance and advance our understanding of why different forms of pretense may produce different cognitive consequences.

To examine whether representational hierarchical complexity and embodiment may help explain variation in children’s executive function performance, the present study employed a 2 (representational hierarchical complexity: primary vs. secondary representation) × 2 (embodiment: embodied vs. disembodied condition) between-subjects design to examine differences in the immediate effects of a single pretense session on children’s executive function. Whereas executive function interventions typically involve repeated activities over several weeks and aim to produce relatively enduring changes in executive function capacity (Thibodeau-Nielsen et al., 2020a), single-session paradigms address a complementary question: whether a brief activity can temporarily alter the recruitment or expression of executive control in a subsequent task. There are two theoretical grounds for examining such immediate effects. First, children’s executive function performance is sensitive to their momentary cognitive and physical states (Diamond, 2013) and may therefore change over relatively short periods in response to brief activities. Second, from the perspective of the Iterative Reprocessing model, as discussed above, engagement in pretense may elicit additional reflective reprocessing, which may, in turn, temporarily strengthen top-down control over behavior (Zelazo, 2015). Supporting the plausibility of such short-term modulation, previous single-session studies have documented immediate changes in executive function performance following brief physical exercise (Naderi et al., 2019), pretense (R. E. White & Carlson, 2021), and video viewing (Kostyrka-Allchorne et al., 2019). Accordingly, the present study employed an executive function pre-test–single pretense session–immediate executive function post-test design to examine potentially transient and process-specific changes in executive function performance. First graders were selected because their representational capacity is sufficiently mature to pass second-order theory of mind tasks (Miller, 2009), which provides a cognitive basis for participating in complex hierarchical representation (Zelazo et al., 2003).

The study also considered potential confounding factors and examined whether representational hierarchical complexity and embodiment interact in influencing children’s executive function performance. Given that children benefit more from training at an appropriate level of difficulty, and that childhood is a critical period for the development of representational skills (Miller, 2009), children with higher representational skills may benefit more from the activity (R. E. White & Carlson, 2016). Therefore, this study used the participants’ level of second-order theory of mind as a control variable. Given the prevalence of positive affect in play (Krasnor & Pepler, 1980; Russ & Dillon, 2011) and its documented role in enhancing executive function performance (Yang & Yang, 2014), it is plausible that improvements in children’s performance on executive function tasks stem from enhanced affective states, rather than from play itself. Consequently, differences in affective states across the groups were also analyzed in this study. Furthermore, to account for potential confounding, this study collected data on participants’ gender, age, and family socioeconomic status for consideration as potential covariates in subsequent analyses. Finally, to further examine the relationship between representational hierarchical complexity and embodiment, it is necessary to consider their potential interaction. Higher representational complexity may provide richer psychological content and more opportunities for reflection, especially when external embodied support is limited, fostering the development of executive function. Conversely, in contexts with lower representational demands, greater embodiment—through enhanced bodily and environmental interaction—may compensate for simpler mental representations and may be associated with greater psychological involvement, self-reflection, and behavioral regulation. When both complexity and embodiment are high, they may operate synergistically, potentially contributing to greater engagement in pretense and cognitive regulation and, in turn, to better executive function performance.

Drawing on the theoretical and empirical considerations presented above, it was hypothesized that children in the secondary representation condition would exhibit superior executive function performance compared to those in the primary representation condition, and that children in the embodied condition would outperform those in the disembodied condition. Given the complexity of the potential interplay between representational hierarchical complexity and embodiment, no directional hypothesis was formulated for their interaction; instead, the interactive effects of these two factors were examined as an open research question.

## 2. Materials and Methods

### 2.1. Participants

The participants were 142 first-grade children (all Han ethnicity) from a primary school in downtown Changchun, Jilin Province, China, with typical developmental trajectories. Following the pre-test assessment, participants were randomly allocated to four experimental conditions—Primary Representation + Disembodied Condition, Primary Representation + Embodied Condition, Secondary Representation + Disembodied Condition, and Secondary Representation + Embodied Condition—using a restricted randomization procedure with prespecified allocation counts (35, 36, 36, and 35 participants for the four conditions, respectively). The allocation sequence was generated by a research coordinator who was not involved in participant assessment or intervention delivery. Specifically, the coordinator created a list of condition labels corresponding to the prespecified group sizes and randomly permuted these labels using a computer-generated procedure. Participants were assigned sequentially to conditions according to the resulting allocation sequence by the research coordinator after completion of the pre-test assessment. Before the intervention and post-test, 11 children were absent from school and therefore did not participate in either the pretense activity or the post-test assessment, with 3, 3, 2, and 3 children lost from the four conditions, respectively. ANOVA indicated no significant differences in pre-test executive function scores between these 11 children and the remaining participants. Accordingly, the final group sizes were 32, 33, 34, and 32, respectively, yielding a final sample of 131 participants. The corresponding numbers of boys in the four groups were 21 (65.6%), 20 (60.6%), 18 (52.9%), and 11 (34.4%), respectively. The final sample ranged in age from 6.33 to 9.17 years (*M*_age_ = 6.90, *SD* = 0.36) and consisted of 70 boys (53.44%) and 61 girls (46.56%). The upper age bound of 9.17 years corresponded to a single participant who was identified as a univariate age outlier based on the sample age distribution. As a sensitivity analysis indicated that the overall pattern and interpretation of the findings were not materially affected by this participant’s inclusion or exclusion, the participant was retained in the primary analyses. Sample size estimation for power analysis was based on the effect size (f = 0.5) reported in the most comparable prior study (R. E. White & Carlson, 2021). To conservatively ensure adequate power (80%, α = 0.05) for detecting the main effects of representational hierarchical complexity, embodiment, and their interaction, we adopted a medium effect size estimate (f = 0.25; Cohen, 1992). A power analysis conducted using G*Power 3.1 indicated that a total of 128 participants would be required. Trial type (e.g., congruent vs. incongruent; shift vs. non-shift) was also planned as a within-subjects factor in the analyses of inhibition and shifting. A separate power analysis suggested that detecting the three-way interaction among trial type, representational hierarchical complexity, and embodiment would require at least 48 participants. Participants’ socioeconomic status (SES) was calculated as the average Z-scores of the father’s and mother’s education levels and occupations (the basic information of the child carers is shown in Table 1). After the experiment, participants received stickers and brief verbal praise (e.g., “Great job playing today!”) as rewards. Informed consent was obtained from the children, parents, and the school.

### 2.2. Experimental Design and Procedure

This study employed a 2 (representational hierarchical complexity: primary representation vs. secondary representation) × 2 (embodiment: embodied condition vs. disembodied condition) between-subjects design, with executive function as the dependent variable. Each participant was assigned to one of the four experimental groups (see Table 2 for group differences). The experiment was conducted in the laboratory across two time points spaced two weeks apart. This schedule aimed to reduce participant fatigue, ensure data quality, and maintain children’s attention. Each time point lasted approximately 30 min.

**Figure 1:**
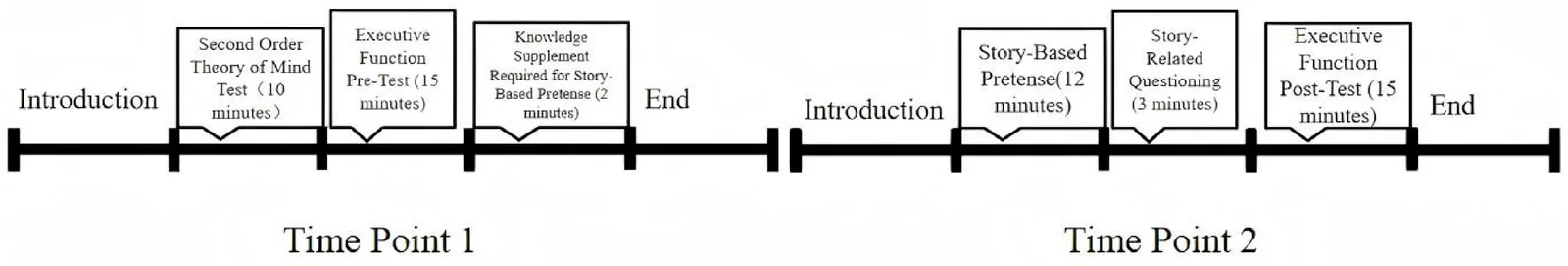
Flow chart of the experiment.

At Time Point 1, participants completed a second-order theory of mind task and a pre-test of executive function, followed by a knowledge supplement phase (see Figure 1). At Time Point 2, participants first engaged in a story-based pretense activity, immediately followed by a comprehension check of story content, and finally completed a post-test of executive function (see Figure 1).

To assess children’s affect during the pretense activity at Time Point 2, the experimenter rated each child’s affective state using a single-item, five-point observational scale based on R. E. White and Carlson (2021). The rating was based on the experimenter’s overall impression of the child’s affective state during the activity (see Supplementary Materials).

During the knowledge supplement phase (Time Point 1; for a full introduction of the story, see the “Operationalization of Representational Hierarchical Complexity” section below), to ensure all children understood the core premise of the story prior to engaging in pretense (i.e., that foxes and cranes are suited to eating from plates or bottles, respectively), the experimenter presented images of a fox, a crane, a plate, and a bottle, and asked: “Should the fox eat from a plate or a bottle? What about the crane? Why?” If the child responded incorrectly, the experimenter provided corrective feedback and explained the relevant knowledge until the child could correctly restate it. This phase was independent of the subsequent pretense activity and did not involve specific story content.

In the pretense intervention phase (Time Point 2), participants first watched a 2-min-and-50-s customized story video (see Supplementary Materials for the full script). After viewing, the participant and experimenter engaged in pretense based on the video. The operationalization of representational hierarchical complexity and the manipulation of embodiment are detailed in Table 2 and in the corresponding subsections below.

In the story comprehension phase (immediately following pretense at Time Point 2), the experimenter assessed children’s understanding of either first-order or second-order mental states, depending on their representational complexity condition. As the primary and secondary representation groups required children to comprehend characters’ first-order and second-order mental states respectively (see the subsection on the operationalization of representational hierarchical complexity below for details), verifying this understanding was a necessary prerequisite for examining the effect of representational hierarchical complexity on executive function. Participants answered 12 questions: 8 assessing memory of objective story facts (e.g., “What did the crane use to invite the fox to dinner?”), and 4 assessing understanding of the characters’ mental states (e.g., “When the crane said it would invite the fox to dinner, did the fox believe it?”). The mental state questions corresponded to the assigned representation group (first-order for primary representation, second-order for secondary representation). Because factual understanding was essential for interpreting mental states, the experimenter only proceeded to the 4 mental state questions after confirming that the child correctly answered all 8 factual questions. All children were able to answer the factual questions correctly. Each question was scored on a 3-point scale (0 = entirely incorrect; 1 = partially correct; 2 = entirely correct).

#### 2.2.1. Operationalization of Representational Hierarchical Complexity

The study used a fairy tale to develop two matched story versions (identical in word count and sentence structure; see Supplementary Materials). All participants watched one version at Time Point 2 and then engaged in story-based pretense with the experimenter (the child played the fox, and the experimenter played the crane). The instruction was: “Now you are the fox, and I am the crane. Let’s listen to a story about the fox and the crane, and then act it out together, okay?” Both story versions involved reciprocal invitations between the fox and the crane. Their construction followed the methodological logic of established story-based mentalizing paradigms, in which mentalizing requirements are varied while non-target characteristics of the story materials are controlled as far as possible (Fletcher et al., 1995; Kobayashi et al., 2008; Saxe & Powell, 2006; S. White et al., 2009). For example, S. White et al. (2009) constructed different Strange Story sets to systematically vary the extent to which mentalizing was required while separating this requirement from more general features of story processing. Following this logic, the two versions in the present study used the same characters and reciprocal-invitation structure and were identical in word count and sentence structure. The critical difference concerned the structure of the mental-state relations that children needed to represent: the primary representation version required representing a character’s intention, whereas the secondary representation version additionally required representing another character’s belief about that intention. Thus, the two versions provided comparable story contexts in which the degree of representational embedding could be varied while major surface linguistic features were held constant.

Within the IR framework, representational hierarchical complexity refers to the degree of embedding within the rule system that must be represented and maintained in working memory (Zelazo et al., 2003; Zelazo, 2015). Lower-order rules take the form of conditional relations linking antecedent conditions to consequences. Through reflection, such rules can be considered in relation to one another and subordinated to a higher-order rule that specifies the conditions under which particular lower-order rules should be selected and applied. Accordingly, representational hierarchical complexity increases as additional degrees of embedding are introduced into the rule system. On this basis, the secondary representation condition was designed to require an additional level of representational embedding relative to the primary representation condition: children needed to represent not only a character’s intention but also another character’s belief about that intention. The latter mental-state relation was therefore nested within the former representational context, increasing the degree of embedding required to represent the story structure. Accordingly, the primary versus secondary representation distinction was used as an operationalization of variation in representational hierarchical complexity in the present study. Table 3 summarizes the representational structure of the two conditions.

In the primary representation condition, the story depicted sincere interactions. The fox invited the crane using a bottle, and the crane hosted the fox using a plate. Both characters acted with genuine intentions. This condition required children to infer first-order mental states (e.g., “The crane truly wanted to invite the fox to dinner”). A primary representation entails inferring a character’s first-order mental state (intention, emotion, or belief) based on overt events without embedding that mental state within another character’s mental-state representation (e.g., “Was the character sincere?”). For example, upon seeing “The fox invited the crane to dinner,” the child must consider “Was the fox sincere?” to infer the first-order mental state “The fox genuinely invited the crane.”

In the secondary representation condition, the story involved deceptive interactions. The fox offered food using a plate, and the crane reciprocated with a bottle—both intended to prevent the other from actually eating. This condition required children to infer second-order mental states, such as one character’s belief about another character’s intention (e.g., “Did the crane believe the fox really wanted to invite him?”). A secondary representation involves an additional level of representational embedding relative to a primary representation. Specifically, children must represent character A’s mental state as the content of character B’s belief about that mental state (e.g., “Does the crane believe the fox?”). In this study, children in the secondary representation condition needed to process characters with hidden intentions and represent how another character understood or misunderstood those intentions.

#### 2.2.2. Manipulation of Embodiment

In both the embodied and disembodied conditions, the child and the experimenter sat together to watch the story video. The story included five fixed episodes (see Supplementary Materials). After each scene, they engaged in corresponding story-based pretense (identical instruction in both conditions: “Now let’s act out this part of the story, okay?”).

In the disembodied condition, the child and experimenter used small puppets representing the fox and the crane, along with matching miniature props (plate and bottle) to act out the scenes. For instance, when enacting the fox offering food in a bottle, the child manipulated the fox puppet to hand a small bottle to the experimenter’s crane puppet.

In the embodied condition, both the child and experimenter wore costumes of the fox and crane, respectively, and used full-sized real props to act out the story. For example, when enacting the fox offering food in a bottle, the child (dressed as the fox) treated themselves as the fox character and physically handed a real bottle to the experimenter (dressed as the crane).

### 2.3. Measures

#### 2.3.1. Executive Function

The measurement tasks for the three core components of executive function (inhibition, working memory, and shifting) were identical in both pre- and post-test phases and were administered on a touchscreen device. The duration of the tasks was approximately 4 min for inhibition, 3 min for working memory, and 8 min for shifting. To control for the impact of order effects, the order of task administration was counterbalanced.

**Figure 2:**
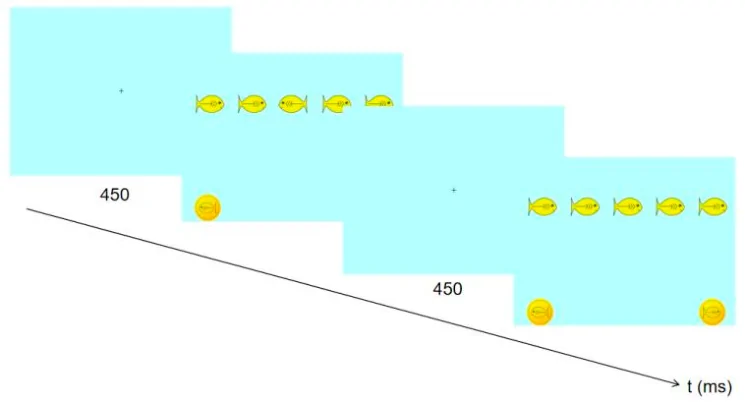
Flowchart of the fish flanker task.

Inhibition—Fish Flanker Task. This experiment employs a widely used task to evaluate children’s inhibitory control (Rueda et al., 2004). Participants are seated in front of a screen and instructed to press either the “left” or “right” key, depending on the direction in which a central fish image is oriented. Each trial display consisted of a central target fish flanked by two distractor fish on each side, totaling five fish per display (see Figure 2). In congruent trials, the side fish matched the direction of the central fish, while in incongruent trials, they pointed in the opposite direction. Before the formal test phase, participants completed a practice phase consisting of six trials: three congruent and three incongruent. To proceed to the formal phase, children were required to respond correctly on at least four out of the six practice trials. If this criterion was not met, the practice phase was repeated once. Children who failed to pass the practice phase after two attempts were considered unable to understand the task instructions and did not proceed to the formal testing phase. The proportion of incongruent trials was 0.33, consisting of 12 incongruent and 24 congruent trials. Each subsequent trial begins immediately after the participant responds. Given the near-ceiling accuracy observed in the Fish Flanker Task (M = 0.99, SD = 0.04), reaction time was selected as the primary outcome measure to better capture individual differences in inhibitory control. Accordingly, inhibitory control was indexed by participants’ response times on incongruent trials, with slower responses reflecting weaker inhibition. Using formal-phase trials only, Spearman–Brown-corrected odd–even split-half reliabilities for congruent- and incongruent-trial reaction times were 0.965 and 0.967 at pre-test and 0.973 and 0.951 at post-test, respectively.

**Figure 3:**
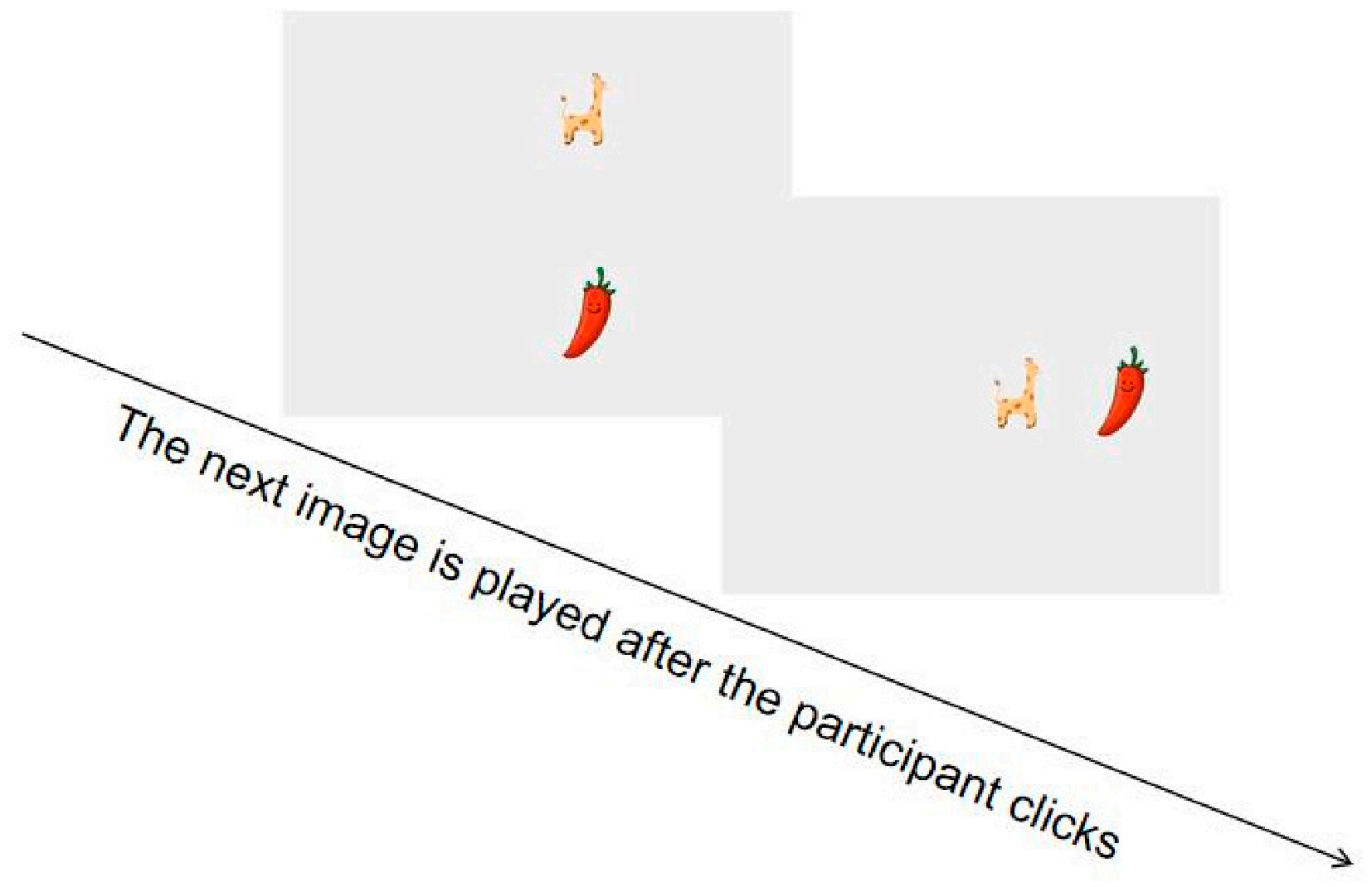
Flowchart of the self-ordered pointing task.

Working Memory—Self-Ordered Pointing Task. In this task, several images are presented on the device screen. The images depict objects commonly encountered in children’s daily lives, such as animals, plants, household items, and vehicles. Participants are first required to select any one of the images by clicking on it. In subsequent trials, participants must select an image that they have not previously clicked. The task proceeds according to this rule until the participant either makes an error by selecting a previously chosen image or successfully selects all images displayed on the screen. A set of trials presenting the same number of images is defined as a trial block, which consists of several consecutive trials. For example, when two images are presented on the screen (see Figure 3), the trial block includes two trials: in the first trial, the participant may select any image (e.g., a giraffe); in the second trial, they must select the other image that has not yet been chosen (e.g., a carrot). If the participant correctly selects images in both trials, they complete that trial block. Each trial block allows for two attempts; if the first attempt is incorrect, the participant is given one more opportunity to complete the block correctly. The task begins with trial blocks containing two images and gradually increases the number of images in each block by one to increase task difficulty. For instance, after completing the two-image trial block, the system automatically advances to a trial block containing three images, and so on. The participant’s working memory span is measured by the number of images presented in the last successfully completed trial block (Hongwanishkul et al., 2005). Working memory capacity is thus reflected by the working memory span, with higher values indicating better working memory ability. For the task, scores were approximately symmetrically distributed at both pre-test (M = 5.40, SD = 1.30, skewness = −0.03, kurtosis = −0.59) and post-test (M = 5.74, SD = 1.36, skewness = 0.07, kurtosis = −0.36).

**Figure 4:**
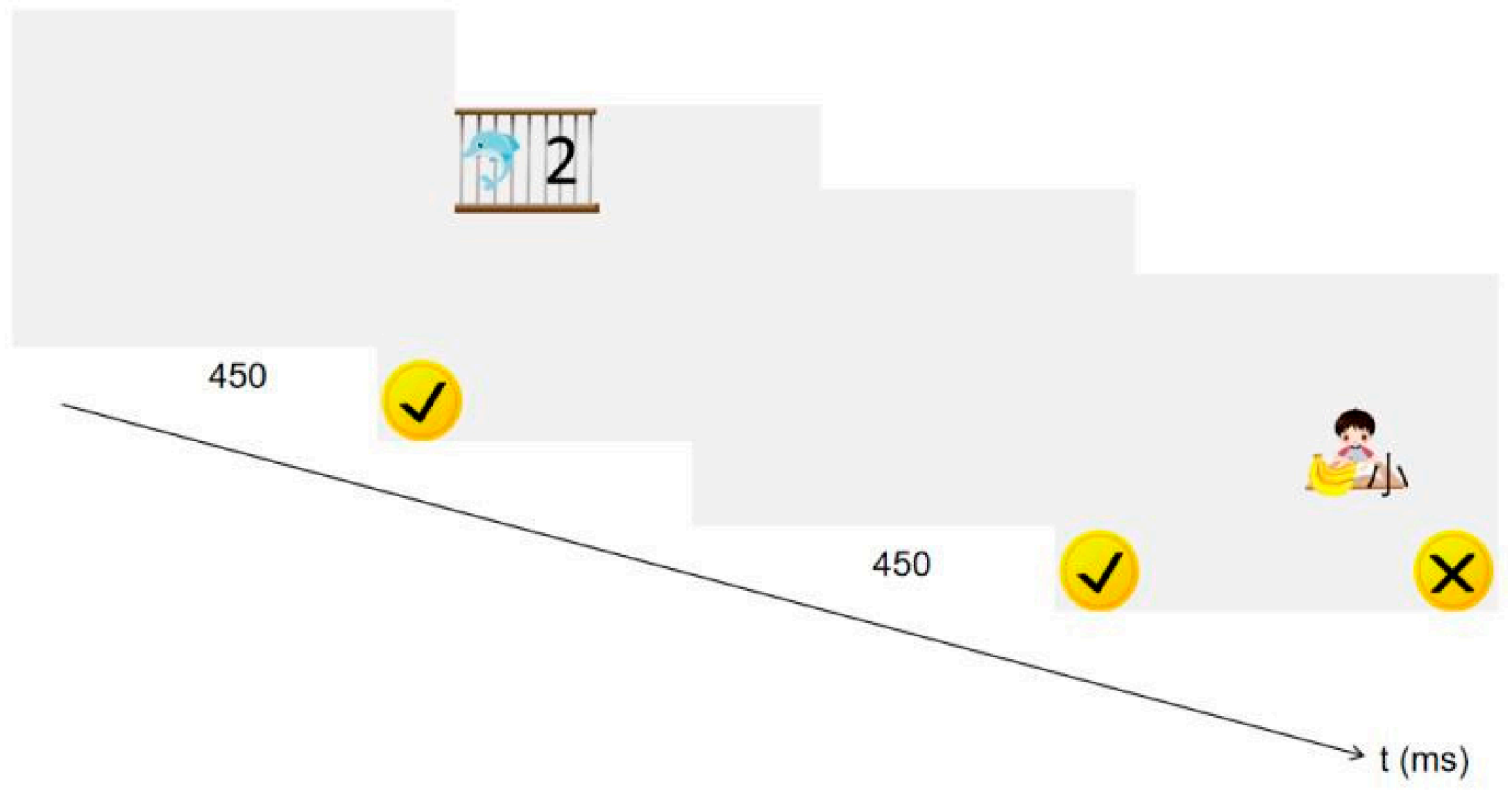
Flowchart of the picture–symbol task.

Shifting—Picture–Symbol Task. This task was adapted from the number–letter task (Miyake et al., 2000; Lee et al., 2012). On each trial, a compound stimulus composed of an image (either an animal or a fruit) and a symbol (either a digit or a Chinese character) was presented in one of four screen quadrants (Quadrant I: top right; Quadrant II: top left; Quadrant III: bottom left; Quadrant IV: bottom right) and remained on screen until a response was made. The image was always displayed to the left of the symbol. When the stimulus appeared in Quadrants I or II (i.e., the upper half of the screen), children were required to judge whether the image was an animal. When the stimulus appeared in Quadrants III or IV (i.e., the lower half of the screen), they were asked to judge whether the symbol was a digit. Each presentation of the compound stimulus was accompanied by an auditory cue asking either “Animal?” or “Digit?” to reinforce the relevant task. The location of the stimulus alternated between the upper (image judgment) and lower (symbol judgment) halves of the screen across trials. After responding, children proceeded to the next trial. Prior to the formal task, children completed a practice phase consisting of four trials: two animal-judgment trials and two digit-judgment trials. To proceed to the formal task, children were required to respond correctly on at least three of the four practice trials. Those who did not meet this accuracy criterion were given a second opportunity to complete the same practice phase. If a child failed to meet the criterion after two practice attempts, the task was terminated, and the child was not included in the formal phase, as this was taken to indicate insufficient understanding of the task rules. This task required participants to switch between two task rules: judging image content and judging symbol content (see Figure 4). Task-switching ability was assessed using accuracy on switch trials, based on findings that accuracy is more sensitive than response time in capturing individual differences in this task (Lee et al., 2012). Higher accuracy on switch trials indicated better switching performance. The proportion of switch trials was 0.48, consisting of 32 switch trials and 34 non-switch trials. Using formal-phase trials only, Spearman–Brown-corrected odd–even split-half reliabilities for switch- and non-switch-trial accuracy were 0.640 and 0.757 at pre-test and 0.755 and 0.804 at post-test, respectively.

#### 2.3.2. Second-Order Theory of Mind

This study assessed participants’ second-order theory of mind using the ice cream task (Perner & Wimmer, 1985). Second-order theory of mind involves the ability to understand what one person thinks about another person’s thoughts—that is, reasoning about nested beliefs. This task specifically requires such reasoning by presenting a scenario in which one character must hold a belief about another character’s belief.

The story was presented to participants via a video, detailing the following sequence of events: 1. In a park, Xiao Hong asks the ice cream vendor to wait while she goes home to get money, and the vendor agrees. 2. After Xiao Hong leaves, Xiao Bai asks the vendor where he is going. The vendor explains that, due to few customers in the park, he will sell ice cream at the shop entrance, prompting Xiao Bai to go home. 3. On his way, the vendor passes by Xiao Hong’s house, where Xiao Hong sees him from the window and inquires about his destination. The vendor informs her that he is heading to the shop entrance to sell ice cream, and Xiao Hong expresses her intention to buy ice cream there. 4. Later, Xiao Bai decides to find Xiao Hong, but Xiao Hong’s mother tells him that she has already gone to buy ice cream.

After the first viewing of the video, participants were asked to self-assess their understanding of the events, recall the facts, and describe the dialogues. The video was then played a second time, and after viewing, participants were asked whether Xiao Bai would go to the park or the shop to find Xiao Hong, and to explain their reasoning.

To correctly answer this question about where Xiao Bai will look for Xiao Hong (the park or the shop), participants must infer Xiao Bai’s belief about Xiao Hong’s location. Importantly, this requires reasoning about what Xiao Bai thinks Xiao Hong believes—a classic example of second-order theory of mind. A correct answer with a valid reason demonstrated the participant’s ability to track nested mental states and was scored 2 points; a correct answer without reasoning was scored 1 point; an incorrect answer was scored 0 points. Higher scores thus indicate a higher level of second-order theory of mind.

## 3. Results

### 3.1. Preliminary Analyses

All executive function (EF) measures were screened for missing data and outliers. In the Fish Flanker task, trials with incorrect responses were excluded at the trial level. Additionally, for each participant, trials in both congruent and incongruent conditions that deviated by more than two standard deviations from their condition-specific mean were removed (proportion of outliers ≤11.11%). At the individual level, extreme values in key indicators (e.g., mean reaction time, accuracy, working memory span) were winsorized by replacing values beyond ±2 SD from the sample mean with the cutoff values. This approach preserves variance while minimizing bias from trimming (proportion of outliers ≤7.63%). Due to poor performance or data loss in specific tasks, some participants were excluded from the relevant analyses: one child responded incorrectly on all incongruent trials in the Fish Flanker task, one child’s data in the Self-Ordered Pointing task were lost due to technical failure, and two children failed to pass the practice phase of the Picture–Symbol task. Descriptive statistics for pre- and post-test variables are shown in Table 4.

To examine baseline equivalence, one-way ANOVAs were conducted to assess between-group differences in gender, age, socioeconomic status (SES), second-order theory of mind, and pre-test scores on the three EF components. All ANOVAs in the present study were performed using SPSS 26. Results indicated no significant group differences in age (*F*(3, 127) = 0.99, *p* = 0.399), SES (*F*(3, 127) = 0.01, *p* = 0.998), second-order theory of mind (*F*(3, 127) = 0.78, *p* = 0.505), or pre-test EF components (inhibition: *F*(3, 127) = 1.59, *p* = 0.196; working memory: *F*(3, 127) = 1.26, *p* = 0.290; shifting: *F*(3, 127) = 0.25, *p* = 0.859). The between-group difference in gender did not reach the conventional level of statistical significance (coded as 1 = male, 2 = female; *F*(3, 127) = 2.49, *p* = 0.064, *η*^2^_p_ = 0.055, 90% CI [0.000, 0.114]). Thus, the groups were generally comparable on baseline characteristics, although descriptive variation in gender distribution was observed across groups, as reflected in the group-specific distributions reported in Section 2.1. Affect assessed during the pretense activity also did not differ significantly across groups, *F*(3, 127) = 1.08, *p* = 0.361. Given previous evidence indicating associations between gender and executive function during kindergarten and the early years of primary school (Montroy et al., 2016; Ribeiro et al., 2021; Silverman, 2021; Zysset et al., 2018), gender was considered a theoretically justified potential covariate. However, its formal inclusion as a covariate was determined after inspection of the baseline between-group comparisons. Considering both the theoretical relevance of gender to executive function and the observed descriptive variation in gender distribution across groups, gender was included as a covariate in subsequent analyses to account for potential gender-related variation in executive function performance. As a sensitivity analysis, the primary analyses were also repeated without gender as a covariate to assess whether its inclusion affected the overall pattern of results. In addition, as discussed in the introduction, children at different levels of representation may benefit differently from engaging in pretense. Therefore, to examine the intervention effects on children’s executive function, analyses were conducted both with and without controlling for second-order theory of mind.

To assess children’s understanding of characters’ mental states during the pretense activity, their responses to four questions at Time 2 were analyzed. Each question was worth two points, with a maximum total score of 8. Results showed that 84.0% of children received full marks, 3.1% scored 6 points, 12.2% scored 4 points, and 0.8% scored 0. The average score was 7.39 (*SD* = 1.49). These findings suggest that, overall, most children were able to accurately interpret the characters’ mental states in the story.

### 3.2. Effects of Representational Hierarchical Complexity and Embodiment on Executive Function

To examine the effects of representational hierarchical complexity and embodiment on children’s executive function, we conducted four ANCOVAs, using mixed-design ANCOVAs for outcomes that included a within-subject factor. Because the study involved one baseline assessment and one immediate post-test, each post-test outcome was analyzed with its corresponding baseline measure entered as a covariate. ANCOVA was preferred to change-score analysis because change scores are susceptible to measurement error and regression to the mean and may not adequately address chance baseline imbalances. By adjusting post-test performance for its association with baseline performance, ANCOVA generally provides more precise and statistically efficient estimates of experimental-condition effects in randomized pre-test–post-test designs (Vickers & Altman, 2001; Senn, 2006; van Breukelen, 2006). A repeated-measures mixed-effects model incorporating both baseline and post-test scores would also have been a valid analytical approach (Wan, 2018). However, ANCOVA was more closely aligned with the present study’s measurement structure and inferential objective. Baseline performance was assessed before the experimental manipulation and was therefore treated as a baseline covariate reflecting individual differences in initial performance, whereas immediate post-test performance served as the outcome for evaluating experimental-condition effects (Wan, 2021). Given the availability of only one baseline and one immediate post-test assessment, ANCOVA provided a parsimonious and readily interpretable estimate of adjusted between-condition differences.

The four post-test dependent variables were reaction time in the inhibition task, working memory span, accuracy in the shifting task, and the executive function composite score, which was computed as the mean of the standardized scores for incongruent reaction time (with the sign reversed), working memory span, and shifting accuracy. To account for multiple testing across executive function outcomes, Bonferroni correction was applied separately to the main effect of representational hierarchical complexity, the main effect of embodiment, and their interaction. For each effect, results were considered statistically significant at *p* < 0.05 with Bonferroni correction; that is, 0.05/4 = 0.0125 for the four executive function outcomes. Trial-type effects and interactions involving trial type were not included in this across-outcome correction because trial type was not defined equivalently across all four outcomes. The across-outcome correction was separate from the Bonferroni correction applied to the four follow-up simple-effect comparisons within the inhibition outcome, for which the corrected significance threshold was also *p* < 0.0125 (0.05/4).

**Figure 5:**
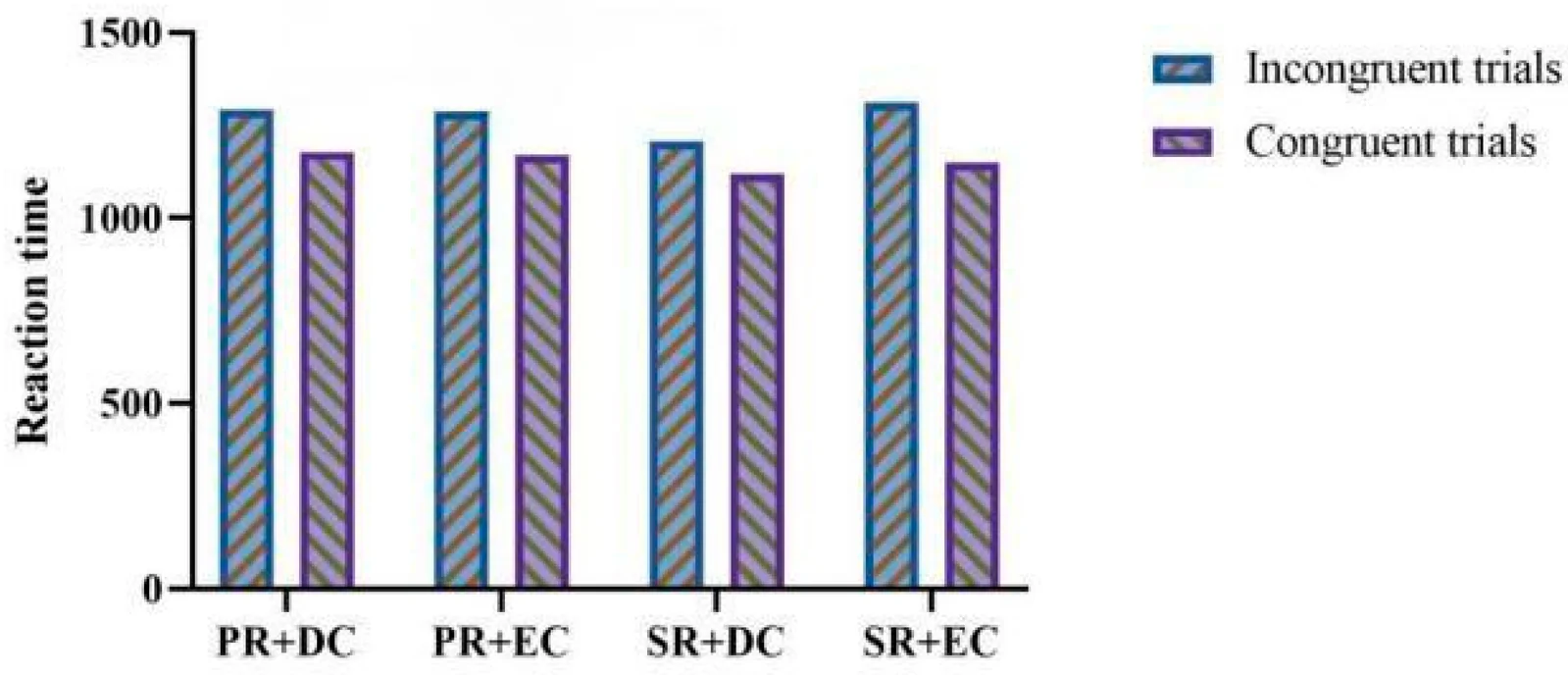
The effects of representational hierarchical complexity and embodiment on inhibitory control. Note. PR = Abbreviation for primary representation; SR = Abbreviation for secondary representation; DC = Abbreviation for disembodied condition; EC = Abbreviation for embodied condition.

For the inhibition task, a 2 (Trial Type: congruent vs. incongruent) × 2 (Representational Hierarchical Complexity: primary vs. secondary) × 2 (Embodiment: disembodied vs. embodied) mixed-design ANCOVA was conducted, controlling for gender and baseline congruent and incongruent RTs. Prior to the analysis, the assumptions of homogeneity of variance, homogeneity of regression slopes, and normality of residuals were evaluated. Levene’s tests were nonsignificant for both post-test incongruent-trial RTs, *F*(3, 126) = 1.154, *p* = 0.330, and post-test congruent-trial RTs, *F*(3, 126) = 0.251, *p* = 0.861, indicating that the homogeneity-of-variance assumption was satisfied. None of the interactions between the experimental factors and covariates reached statistical significance (all *ps* ≥ 0.114), indicating that the homogeneity-of-regression-slopes assumption was not violated. Tests of residual normality showed that the Kolmogorov–Smirnov tests were nonsignificant for the standardized residuals of both incongruent-trial RTs, *D*(130) = 0.073, *p* = 0.088, and congruent-trial RTs, *D*(130) = 0.064, *p* = 0.200. However, the corresponding Shapiro–Wilk tests were significant, *W*(130) = 0.973, *p* = 0.010, and *W*(130) = 0.977, *p* = 0.027, respectively. Further examination of the histograms, Q–Q plots, and skewness and kurtosis indices indicated that the departures from normality were largely confined to the distributional tails and were minor overall. Given the robustness of ANOVA to violations of normality (Levy, 1980; Rutherford, 2011) and the absence of substantial deviations or extreme residuals, these minor departures from residual normality were not considered likely to materially affect the statistical inferences. Results revealed no significant main effects of trial type (*F*(1,123) = 1.61, *p* = 0.207, *η*^2^_p_ = 0.013, 90% CI [0.000, 0.064]), representational hierarchical complexity (*F*(1,123) = 1.19, *p* = 0.278, *η*^2^_p_ = 0.010, 90% CI [0.000, 0.057]), or embodiment (*F*(1,123) = 1.85, *p* = 0.176, *η*^2^_p_ = 0.015, 90% CI [0.000, 0.068]). Similarly, neither of the two-way interactions involving trial type (trial type × complexity: *F*(1, 123) = 0.02, *p* = 0.900, *η*^2^_p_ = 0.000, 90% CI [0.000, 0.007]; trial type × embodiment: *F*(1, 123) = 0.02, *p* = 0.886, *η*^2^_p_ = 0.000, 90% CI [0.000, 0.007]) nor the three-way interaction (*F*(1, 123) = 3.64, *p* = 0.059, *η*^2^_p_ = 0.029, 90% CI [0.000, 0.092]) was significant. The interaction between representational hierarchical complexity and embodiment reached significance at the conventional uncorrected threshold, *F*(1,123) = 5.08, *p* = 0.026, *η*^2^_p_ = 0.040, 90% CI [0.003, 0.109]. However, this effect did not meet the Bonferroni-corrected significance threshold of *p* < 0.0125 across the four executive function outcomes. To characterize the pattern underlying this outcome-specific interaction, four follow-up simple-effect comparisons were evaluated using the Bonferroni-corrected significance threshold of *p* < 0.0125. As illustrated in Figure 5, in the disembodied condition, children in the secondary representation group responded faster (*M* = 1184.13, *SE* = 30.06) than those in the primary representation group (*M* = 1288.04, *SE* = 31.48), *F*(1,123) = 5.73, *p* = 0.018, *η*^2^_p_ = 0.045, 90% CI [0.004, 0.116]; however, this difference did not meet the Bonferroni-corrected significance threshold. Within the primary representation group, children in the embodied condition responded faster (*M* = 1175.49, *SE* = 30.83) than those in the disembodied condition (*M* = 1288.04, *SE* = 31.48), *F*(1,123) = 6.48, *p* = 0.012, *η*^2^_p_ = 0.050, 90% CI [0.006, 0.124], and this difference remained significant after the Bonferroni correction. Thus, only the simple effect of embodiment within the primary representation group met the locally corrected significance threshold. These follow-up comparisons were used to characterize the pattern of the outcome-specific interaction, which itself did not survive the across-outcome Bonferroni correction. This pattern remained unchanged when second-order theory of mind was added as a covariate and, separately, when gender was omitted as a covariate.

For working memory span, a 2 (Representational Hierarchical Complexity) × 2 (Embodiment) between-subjects ANCOVA was conducted, controlling for gender and baseline working memory span. Prior to conducting the ANCOVA, the assumptions of homogeneity of variance, homogeneity of regression slopes, and normality of residuals were evaluated. Levene’s test was nonsignificant, *F*(3, 126) = 0.513, *p* = 0.674, indicating that the homogeneity-of-variance assumption was satisfied. None of the interactions between the experimental factors and the covariates reached statistical significance (all *ps* ≥ 0.056), indicating that the homogeneity-of-regression-slopes assumption was not violated. Both the Shapiro–Wilk and Kolmogorov–Smirnov tests of the standardized residuals were nonsignificant, *W*(130) = 0.987, *p* = 0.279, and *D*(130) = 0.047, *p* = 0.200, respectively. The histogram was approximately symmetrical, and the residuals closely followed the reference line in the Q–Q plot, with only minor deviations in the tails. Taken together, these results indicated that the assumptions of homogeneity of variance, homogeneity of regression slopes, and normality of residuals were satisfied for the working memory task. No significant main effects or interaction were observed (complexity: *F*(1,124) = 0.08, *p* = 0.776, *η*^2^_p_ = 0.001, 90% CI [0.000, 0.024]; embodiment: *F*(1,124) = 2.98, *p* = 0.087, *η*^2^_p_ = 0.023, 90% CI [0.000, 0.083]; interaction: *F*(1,124) = 0.02, *p* = 0.893, *η*^2^_p_ = 0.000, 90% CI [0.000, 0.007]). These findings remained unchanged when second-order theory of mind was added as a covariate and, separately, when gender was omitted as a covariate.

For shifting accuracy, a 2 (Trial Type: shift vs. non-shift) × 2 (Representational Hierarchical Complexity) × 2 (Embodiment) mixed-design ANCOVA was conducted, controlling for gender and baseline accuracy in both trial types. Prior to conducting the ANCOVA, the assumptions of homogeneity of variance, homogeneity of regression slopes, and normality of residuals were evaluated. Levene’s tests were nonsignificant for post-test accuracy on both A and B trials, *F*(3, 125) = 0.164, *p* = 0.921, and *F*(3, 125) = 0.296, *p* = 0.828, respectively, indicating that the homogeneity-of-variance assumption was satisfied. None of the interactions between the experimental factors and the covariates reached statistical significance (all *ps* ≥ 0.162), indicating that the homogeneity-of-regression-slopes assumption was not violated. For non-switch trials, both the Kolmogorov–Smirnov and Shapiro–Wilk tests of the standardized residuals were nonsignificant, *D* = 0.056, *p* = 0.200, and *W* = 0.983, *p* = 0.108, respectively, indicating that the residuals were approximately normally distributed. For switch trials, however, both tests were significant, *D* = 0.097, *p* = 0.005, and *W* = 0.970, *p* = 0.006, respectively, indicating a statistically significant departure from normality. Nevertheless, visual inspection of the histogram and Q–Q plot showed that the deviation was largely confined to the distributional tails. Moreover, the standardized residuals ranged from −2.74 to 2.66, with no extreme residuals identified. Given the robustness of ANOVA to violations of normality (Levy, 1980; Rutherford, 2011), and because the deviation was confined primarily to the tails and no extreme residuals were observed, the departure was considered limited in magnitude and did not constitute a serious violation of the normality assumption. It was therefore unlikely to have materially affected the statistical inferences. Results revealed no significant main effects or interactions (trial type: *F*(1,122) = 0.65, *p* = 0.422, *η*^2^_p_ = 0.005, 90% CI [0.000, 0.046]; complexity: *F*(1,122) = 1.20, *p* = 0.276, *η*^2^_p_ = 0.010, 90% CI [0.000, 0.057]; embodiment: *F*(1,122) = 0.06, *p* = 0.800, *η*^2^_p_ = 0.001, 90% CI [0.000, 0.020]; complexity × embodiment: *F*(1,122) = 0.23, *p* = 0.635, *η*^2^_p_ = 0.002, 90% CI [0.000, 0.034]; trial type × complexity: *F*(1,122) = 2.04, *p* = 0.156, *η*^2^_p_ = 0.016, 90% CI [0.000, 0.071]; trial type × embodiment: *F*(1,122) = 0.11, *p* = 0.741, *η*^2^_p_ = 0.001, 90% CI [0.000, 0.027]; and three-way interaction: *F*(1,122) = 0.90, *p* = 0.345, *η*^2^_p_ = 0.007, 90% CI [0.000, 0.051]). These results also remained consistent when second-order theory of mind was added as a covariate and, separately, when gender was omitted as a covariate.

For the executive function composite score, a 2 (Representational Hierarchical Complexity) × 2 (Embodiment) between-subjects ANCOVA was conducted, controlling for gender and baseline composite score. Prior to conducting the ANCOVA, the assumptions of homogeneity of variance, homogeneity of regression slopes, and normality of residuals were evaluated. Levene’s test was nonsignificant, *F*(3, 127) = 1.684, *p* = 0.174, indicating that the homogeneity-of-variance assumption was satisfied. None of the interactions between the experimental factors and the covariates reached statistical significance (all *ps* ≥ 0.081), indicating that the homogeneity-of-regression-slopes assumption was not violated. Both the Kolmogorov–Smirnov and Shapiro–Wilk tests of the standardized residuals were nonsignificant, *D*(131) = 0.055, *p* = 0.200, and *W*(131) = 0.987, *p* = 0.249, respectively. The standardized residuals exhibited only slight negative skewness (−0.394) and modest kurtosis (0.320), and the Q–Q plot showed that most residuals closely followed the expected normal distribution. Taken together, these results indicated that the assumptions of homogeneity of variance, homogeneity of regression slopes, and normality of residuals were satisfied for the executive function composite score. No significant main effects or interaction were found (complexity: *F*(1,125) = 1.47, *p* = 0.228, *η*^2^_p_ = 0.012, 90% CI [0.000, 0.061]; embodiment: *F*(1,125) = 1.07, *p* = 0.303, *η*^2^_p_ = 0.008, 90% CI [0.000, 0.054]; interaction: *F*(1,125) = 0.21, *p* = 0.651, *η*^2^_p_ = 0.002, 90% CI [0.000, 0.032]). These results remained stable when second-order theory of mind was added as a covariate and, separately, when gender was omitted as a covariate.

Taken together, none of the main effects of representational hierarchical complexity or embodiment, nor their interaction, remained significant after the across-outcome Bonferroni correction.

## 4. Discussion

The present study examined whether representational hierarchical complexity and embodiment jointly influenced children’s immediate executive function performance following a single session of story-based pretense. After correction for multiple testing across the executive function outcomes, no main effect or interaction remained statistically significant. Before correction across the executive function outcomes, an interaction between representational hierarchical complexity and embodiment was observed for inhibitory control. Follow-up analyses showed better inhibition performance for embodied than disembodied children within the primary representation condition, a difference that met the Bonferroni-adjusted significance threshold for the follow-up simple-effect comparisons, whereas the advantage of secondary over primary representation within the disembodied condition did not meet this threshold. Because the overall interaction did not remain significant after correction for multiple testing across the executive function outcomes, these condition-specific patterns should be regarded as exploratory rather than confirmatory. Neither representational hierarchical complexity nor embodiment produced significant main effects on inhibition, and no significant main effects or interactions were observed for working memory, task switching, or the executive function composite score. Thus, the null effect on the executive function composite, alongside the absence of robust effects across the individual executive function outcomes, indicates that the present findings do not provide robust evidence that representational hierarchical complexity or embodiment has a broad influence on children’s subsequent executive function performance.

Building on previous research on the relation between pretense and executive function, the present study extended this line of work by examining whether specific characteristics of pretense—representational hierarchical complexity and embodiment—influence children’s subsequent executive function performance. Previous studies have reported associations between children’s engagement in pretense and executive function performance (Metaferia et al., 2020a, 2020b, 2021; Thibodeau-Nielsen et al., 2020b; Van Reet, 2020; R. E. White et al., 2021), as well as short-term and longer-term improvements following pretense activities or interventions (Thibodeau et al., 2016; R. E. White & Carlson, 2016, 2021; R. E. White et al., 2017). Among these studies, R. E. White and Carlson (2021) is particularly relevant to the present study because they examined the immediate effects of story-related pretense on children’s inhibitory control. Specifically, they examined the effects of story content (fantastical vs. realistic) and mode of engagement with the story (pretense vs. a non-pretense activity) on children’s subsequent inhibitory control performance. Story content and its interaction with engagement mode were not significant, whereas children who engaged in story-related pretense showed better inhibitory control than those who participated in the non-pretense activity. The present study built on this work in two respects. First, it broadened the executive function outcomes examined by assessing inhibition, working memory, and task switching rather than inhibitory control alone. Second, while R. E. White and Carlson examined story content and whether children engaged in pretense, the present study focused specifically on variation within pretense by operationalizing representational hierarchical complexity and manipulating embodiment. Drawing on the Iterative Reprocessing model and research on embodied cognition, the study therefore addressed a more specific question concerning whether these representational and embodied characteristics of pretense influence children’s subsequent executive function performance. The results did not provide statistically robust evidence that representational hierarchical complexity, embodiment, or their interaction affected executive function performance after correction for multiple testing across outcomes. Nevertheless, the unadjusted analyses revealed an exploratory interaction between representational hierarchical complexity and embodiment for inhibitory control. Thus, although the present study does not establish that particular forms of pretense are more effective for promoting executive function, it helps refine the question of how specific features of pretense may relate to children’s immediate subsequent executive function performance. Given the single-session design, these findings should not be interpreted as evidence that similar effects would emerge or accumulate over longer-term pretense interventions.

The absence of effects surviving correction for multiple testing places an important constraint on the hypothesized roles of representational hierarchical complexity and embodiment. Specifically, varying these characteristics within a single session of pretense was not sufficient to produce statistically robust differences in children’s subsequent executive function performance in the present sample. This pattern may indicate that the two characteristics, as operationalized here, do not reliably influence executive function either independently or jointly at an immediate timescale. However, the null-corrected results do not establish the absence of any effect. Such effects may be relatively small and therefore difficult to detect reliably following a single session, or they may emerge only through repeated engagement with pretense over a longer period. Against this broader pattern, the unadjusted interaction observed for inhibition should be interpreted cautiously. Given its relatively modest effect size (*η*^2^_p_ = 0.040), its isolation to the inhibition outcome, and its failure to survive correction across executive function outcomes, the possibility that this interaction is a chance finding cannot be excluded. Accordingly, it cannot provide confirmatory evidence that representational hierarchical complexity and embodiment jointly influence inhibitory control. Nevertheless, the direction of this exploratory pattern raises the more specific possibility that the relevance of representational complexity may depend on the degree of embodied support available during pretense. This possibility remains tentative and should be tested directly in independent studies designed to evaluate this interaction.

At the theoretical level, the present findings call for a more cautious interpretation of the proposed roles of representational hierarchical complexity and embodiment. The Iterative Reprocessing model proposes that executive function depends on repeated reflection on information and the construction of increasingly complex representational and rule structures (Zelazo, 2015). From this perspective, pretense involving greater representational hierarchical complexity may provide more opportunities for reflection and reprocessing, thereby supporting the construction, maintenance, and flexible application of behavior-guiding rules. Similarly, research on embodied cognition suggests that bodily engagement and environmental affordances can shape cognitive processing (Foglia & Wilson, 2013; Glenberg, 1997; Glenberg et al., 2013; Stolz, 2015; Wilson, 2002). Greater embodiment may strengthen body–environment integration, enhance situational realism, and increase psychological involvement in the pretend activity, which may in turn support self-reflection, reprocessing, and the regulation of behavior in accordance with imagined rules. However, after correction for multiple testing, the present findings did not provide robust support for the predicted effects of representational hierarchical complexity, embodiment, or their interaction. These findings therefore constrain the empirical predictions derived from these theoretical perspectives. At the same time, the theoretical accounts considered here implicate several cognitive processes that may contribute to these effects, including reflection, iterative reprocessing, rule construction and maintenance, and psychological involvement. The present study, however, was designed to examine whether representational hierarchical complexity and embodiment were associated with differences in subsequent executive function performance rather than to directly assess these underlying processes. Accordingly, the current findings cannot determine whether these processes differed across the experimental conditions despite the absence of detectable differences in executive function performance following a single pretense session. Thus, the present results constrain the predicted behavioral consequences of representational hierarchical complexity and embodiment, while the specific cognitive processes implicated in these theoretical accounts remain to be directly tested. Other accounts of pretense, including sociocultural and creativity-based perspectives, likewise implicate processes such as rule use, flexible representation, and behavioral regulation (Vygotsky, 1978; Bunce & Woolley, 2021; Thibodeau-Nielsen et al., 2020a; Vasilopoulos & Dumontheil, 2024). However, because these processes were not directly manipulated or measured in the present study, the current findings cannot determine their contribution to children’s executive function performance.

Several features of the study may also help explain why robust differences across conditions were not detected. First, the experimental conditions were implemented within a single pretense session. Representational hierarchical complexity and embodiment may influence executive function only after repeated opportunities to engage with and adapt to these demands, rather than producing readily detectable changes after a brief activity. Second, the characteristics and specific demands of the executive function tasks may have limited transfer from the pretense manipulation. For inhibition, although pretense requires children to suppress responses based on immediate reality and act in accordance with an imagined situation, such regulation is embedded in a meaningful and context-rich pretend scenario. By contrast, the Flanker task requires rapid resolution of perceptual response conflict in a highly decontextualized setting. The inhibitory demands involved in pretense may therefore not transfer readily to the type of perceptual conflict control assessed by the Flanker task. For working memory, pretense involving more complex representations may place demands on maintaining information about the imagined situation. However, the working memory task used in the present study required children to keep track of previously selected visual items and avoid selecting them again. Because these demands differ in content and task structure, experience with maintaining information during pretense may not readily transfer to performance on this task. Similarly, task switching in the present study required children to alternate between abstract task rules in response to experimentally specified cues. Although pretense can involve flexible shifts between representations, roles, and imagined situations, these forms of flexibility may not map directly onto the cue-driven switching processes assessed by the task. Consistent with these component-level null findings, no robust effect was detected for the executive function composite score. Third, the use of only one task for each executive function component may have limited construct coverage and sensitivity to small changes. The absence of corrected effects may therefore reflect very small or absent effects, limited sensitivity to such effects, or a combination of these factors.

The present findings also warrant caution in drawing educational implications. Because no effects remained statistically significant after correction for multiple testing across executive function outcomes, the results do not provide sufficient evidence to recommend using the present operationalization of representational hierarchical complexity or manipulating embodiment, either independently or in combination, as a means of improving children’s executive function. The exploratory inhibition pattern may inform hypotheses for future experimental or intervention studies, but it should not be used to guide instructional practice at this stage. Classroom-based research involving repeated pretense activities, classroom-relevant or ecologically valid outcomes, and longer-term follow-up is needed to determine whether systematic variation in these characteristics can produce reliable and educationally meaningful benefits.

The caution warranted in translating these findings into educational practice should also be considered in light of several limitations of the present study. First, regarding intervention duration and frequency, the single-session manipulation used in this study may not capture the effects of repeated or long-term engagement with story structures varying in representational hierarchical complexity and degree of embodiment; future research should examine how these factors function over extended pretense interventions. Second, the present operationalization of representational hierarchical complexity involved some residual construct-level confounding. Although the two story versions were matched on major linguistic features, the primary representation condition involved sincere intentions, whereas the secondary representation condition involved deception, hidden intentions, and misunderstanding of another character’s intentions. These differences may have introduced additional cognitive or affective demands beyond those associated with representational embedding itself. Accordingly, the primary–secondary representation contrast should be understood as an operationalization of representational hierarchical complexity rather than a pure manipulation of this construct. Future research should seek to manipulate the degree of hierarchical embedding while holding qualitative social content more closely constant, thereby allowing representational hierarchical complexity to be examined more precisely. Third, the embodiment manipulation differed simultaneously across several dimensions, including bodily movement, costume use, object size, and the degree of sensory engagement. Consequently, the present design does not permit us to determine which specific component, or combination of components, may be relevant to executive function performance. Future studies should isolate these elements through more tightly controlled manipulations, such as varying bodily movement while holding costumes, objects, and sensory input constant, to clarify the unique and interactive contributions of different components of embodiment. Fourth, the same EF tasks were administered at pre-test and post-test. Although this approach maintained consistency in task instructions, stimulus structure, response requirements, scoring procedures, and overall difficulty across measurement occasions, repeated exposure may have produced practice effects through increased familiarity with the task rules and response procedures (Calamia et al., 2012; Müller et al., 2012). Because all experimental conditions underwent the same assessment procedures, a general practice effect would be unlikely, by itself, to generate systematic between-condition differences. Nevertheless, repeated administration of identical tasks may have influenced the magnitude or sensitivity of pre-test-to-post-test changes. Future research should therefore employ psychometrically validated equivalent parallel forms where available to reduce the potential influence of repeated task exposure. Fifth, although the final sample size exceeded the a priori requirement based on a medium effect size (*f* = 0.25), the study may have had limited statistical power to detect interaction effects smaller than this assumed magnitude. The resulting per-cell sample sizes (*n* = 32–34) also limit the precision of the estimated between-condition differences. This consideration applies to both the exploratory inhibition interaction and the null findings for working memory and task switching. The former should be interpreted cautiously because its magnitude may be estimated imprecisely, whereas the latter should not be taken as evidence that small effects are absent. Future studies with larger samples are needed to obtain more precise effect estimates and provide greater power to evaluate potentially small interaction effects across executive function components. Sixth, the generalizability of the findings is constrained by the homogeneous sample, which consisted exclusively of first-grade Han Chinese children from a single school. The present pattern of results may therefore partly reflect school-specific educational practices, culturally shaped experiences of pretense and embodiment, or developmental characteristics unique to early primary school children. Accordingly, it remains unclear whether the same pattern would emerge across schools, cultural or ethnic groups, and developmental stages. Future research should examine the robustness of these findings using multisite and more culturally and age-diverse samples. Seventh, the present study used a single story-based pretense context, which limits conclusions about whether the effects of representational hierarchical complexity and embodiment would generalize across different forms or thematic contents of pretense. Different themes—such as violent, prosocial, social-normative, cooperative, or fantastical scenarios—may differ in the cognitive and behavioral demands they place on children and may therefore alter the effects of these characteristics on executive function. Future studies could systematically vary pretense content themes while manipulating representational hierarchical complexity and embodiment to determine whether the effects observed under one thematic context generalize to other forms of pretense.

## 5. Conclusions

The present study examined whether representational hierarchical complexity and embodiment influenced children’s immediate executive function performance following a single session of story-based pretense. After correction for multiple testing across executive function outcomes, no main effects or interactions remained statistically significant. Thus, the present findings do not provide robust evidence that representational hierarchical complexity, embodiment, or their combination influences children’s subsequent inhibition, working memory, task switching, or overall executive function performance. Although an interaction between representational hierarchical complexity and embodiment emerged for inhibitory control in the unadjusted analysis, it did not survive multiplicity correction and should therefore be considered exploratory rather than confirmatory.

These findings place an important constraint on the proposed roles of representational hierarchical complexity and embodiment in pretense: varying these characteristics within a single session was not sufficient to produce statistically robust differences in subsequent executive function performance. Future research should examine these characteristics across repeated or longer-term pretense activities, employ more tightly controlled operationalizations of representational hierarchical complexity in which qualitative social content is held more closely constant, use equivalent parallel forms of executive function tasks where available, and evaluate the effects of these characteristics in larger and more diverse samples. Examining whether different forms or thematic content of pretense alter these effects may further clarify the conditions under which representational hierarchical complexity and embodiment are relevant to executive function. Such work is also needed to determine whether the exploratory inhibition pattern is replicable and whether these characteristics of pretense reliably influence executive function under particular conditions.

## Figures and Tables

**Table 1:** Basic information on the children’s guardians.

Variable	Frequency (Mother)	Percentage (Mother, %)	Frequency (Father)	Percentage (Father, %)
Education level				
Primary school and below	6	4.6	3	2.3
Junior high school	15	11.5	21	16.0
High school or secondary school	33	25.2	33	25.2
College	27	20.6	29	22.1
University	28	21.4	25	19.1
Master’s degree or above	1	0.8	0	0
Not reported	21	16.0	20	15.3
Occupation				
Jobless, unemployed and semi-unemployed	25	19.1	7	5.3
Employees in service and manual labour	15	11.5	25	19.1
Employees in transactional work	14	10.7	13	9.9
Self-employed with no or few employees	25	19.1	32	24.4
Owners of large and medium-sized enterprises	0	0	1	0.8
Middle managers of enterprises	6	4.6	5	3.8
Military or police officers	0	0	0	0
Professionals and technicians	17	13.0	21	16.0
National public officials	3	2.3	1	0.8
Not reported	26	19.8	26	19.8

**Table 2:** Content of pretense activities by group.

		Embodiment	
		Disembodied Condition	Embodied Condition
**Representational Hierarchical Complexity**	Primary Representation Group	Listen to a story requiring understanding of the primary representation of a character Use small dolls and mini props for enactment	Listen to a story requiring understanding of the primary representation of a character Use full-sized costumes and props for enactment
	Secondary Representation Group	Listen to a story requiring understanding of the secondary representation of a character Use small dolls and mini props for enactment	Listen to a story requiring understanding of the secondary representation of a character Use full-sized costumes and props for enactment

**Table 3:** Analysis of story representational hierarchical complexity.

	Objective Event	Primary Representation (Embedded Condition: The Fox Either Does or Does Not Want to Invite)	Secondary Representation (Embedded Condition: The Crane Either Believes or Does Not Believe the Fox)
Primary Representation Group	The fox invites the crane for a meal	The fox really wants to invite the crane for a meal	
Secondary Representation Group	The fox invites the crane for a meal	The fox does not really want to invite the crane for a meal	The crane believes the fox genuinely wants to invite them for a meal

**Table 4:** Descriptive statistics of pre-test and post-test variables.

Variable	PR + DC		PR + EC		SR + DC		SR + EC	
	Pre-Test	Post-Test	Pre-Test	Post-Test	Pre-Test	Post-Test	Pre-Test	Post-Test
**Incongruent Trial Reaction Time for Inhibition Task (ms)**	1509.38 ± 539.79	1293.40 ± 313.79	1812.08 ± 722.14	1288.73 ± 353.37	1624.32 ± 607.04	1215.30 ± 237.22	1754.64 ± 593.47	1308.14 ± 409.19
**Working Memory Span**	5.02 ± 1.34	5.86 ± 1.47	5.45 ± 1.15	5.53 ± 1.31	5.47 ± 1.32	5.94 ± 1.48	5.70 ± 1.37	5.65 ± 1.23
**Accuracy of Response in Shifting Trials**	89.66 ± 6.86	89.00 ± 7.92	88.70 ± 9.13	88.49 ± 8.24	89.14 ± 8.18	91.15 ± 8.04	90.43 ± 8.00	92.16 ± 7.23
**Second-Order Theory of Mind**	0.97 ± 0.97	/	1.21 ± 0.96	/	1.24 ± 0.89	/	0.97 ± 1.00	/

Note. PR = Abbreviation for Primary Representation; SR = Abbreviation for Secondary Representation; DC = Abbreviation for Disembodied Condition; EC = Abbreviation for Embodied Condition.

## Data Availability

The raw data supporting the conclusions of this article will be made available by the authors on request.

## Supplementary Materials

The following supporting information can be downloaded at: https://www.mdpi.com/article/10.3390/bs16091495/s1, File S1: Chinese and English versions of the experimental materials, including the pretend play story text The Fox and the Crane’s Invitation, the affective-state rating scale, and post-story questions.

## Abbreviations

The following abbreviations are used in this manuscript:

EF	Executive Function
IR	Iterative Reprocessing
SES	Socioeconomic Status
