# Supplementary material for: Pretense and Executive Function: Testing the Roles of Representational Hierarchical Complexity and Embodiment
Source: Behav Sci (Basel). 2026 Aug 26;16(9):1495. doi: 10.3390/bs16091495 (PMC13603221; doi:10.3390/bs16091495)
Supplement: Supplementary file 1 [file behavsci-16-01495-s001.zip › behavsci-4403122-supplementary.pdf]

File S1
Chinese version of the experimental material (the version used in the experiments of this study)

Pretend Play Story Text: The Fox and the Crane's Invitation

Note: The black text represents the common parts of the story for both the secondary representation group and the primary representation group. The red text indicates content exclusive to the secondary representation group, and the blue text indicates content exclusive to the primary representation group.

第一幕 森林里
场景一：狐狸遇见仙鹤并请它吃饭
旁白：在很久以前，森林里住着一只狐狸和一只仙鹤，有一天，他们在森林里相遇了。
狐狸心想：这不是仙鹤吗？嘻嘻，我今天要戏弄它，它肯定想不到。（我今天请它吃饭，它会很喜欢的。）
于是狐狸说："仙鹤，你是不是饿了呀，你来我家吧，我请你吃饭哦！"
仙鹤心想：这狐狸真好呀！知道我有点饿了，就来请我吃饭。（我正好有点饿了，就去吃一顿吧。）
于是仙鹤说："好呀好呀！"

第二幕 狐狸家
场景二：仙鹤来狐狸家吃饭
旁白：然后，仙鹤和狐狸来到了狐狸家
【狐狸煮好米饭，把饭放在盘子（瓶子）里，然后将盘子（瓶子）放到桌上】
狐狸说："吃吧，亲爱的仙鹤！"
【仙鹤用又长又细的嘴敲着盘子，敲啊敲，什么也没有吃着。（仙鹤用又长又细的嘴伸进瓶子，吃啊吃，一下子就吃完了。）】
狐狸心想：哈哈，我不是真的想请它吃饭，它没想到吧。（我做的饭确实很好吃呀，它全都吃了。）
狐狸说："仙鹤，好吃吗？"
场景三：仙鹤邀请狐狸吃饭
仙鹤心想：我不能用盘子吃饭，狐狸还用盘子请客，它肯定是故意的，为了报复它，我也要戏弄它，它肯定想不到。（狐狸的饭真是好吃，又香又甜我好喜欢，狐狸它人太好了，为了报答它，我也要请它客，它会很喜欢的。）
然后仙鹤说："好吃，今天真是谢谢你了，为了报答你，我也请你吃一次饭吧！"
狐狸心想：我骗了它，它居然还不知道，哈哈，还要请我吃饭呢！（我请它客，它也请我去它家，哈哈，今天真是请对了！）
于是狐狸说："好呀！"

第三幕 仙鹤家
场景四：狐狸到仙鹤家中吃饭

旁白：第二天，狐狸来到了仙鹤家
【仙鹤已经把饭做好，它把饭倒入瓶子（盘子）里，然后把瓶子（盘子）放到桌上】
仙鹤说："亲爱的狐狸，请吃吧！"
【狐狸开始拿着瓶子吃，但它一会儿用嘴巴贴着瓶口，一会儿闻闻瓶子，不管怎么样，也没法吃到瓶子里的东西（狐狸开始端着盘子吃，它一会儿用嘴巴吃里面的肉，一会儿喝喝鱼汤，一会儿工夫，狐狸就把盘里的饭全吃了）】
仙鹤心想：哈哈，我不是真的想请它吃饭，它没想到吧。（我做的饭确实很好吃呀，它全都吃了。）
仙鹤说道："狐狸，好吃吗？"
场景五：狐狸离开仙鹤家
狐狸心想：仙鹤不是真的想请我吃饭呀！我要是能早一点知道就好了！（仙鹤请我吃的饭真的好吃呀！以后我一定要再吃它家的饭！）
【狐狸什么也没说，脸涨得通红，只好饿着肚子走了（狐狸很感谢仙鹤，拍了拍肚子，出来之后回家去了）】

Records

一、儿童在活动中的情绪状态评分：（　　　）
【评分标准】
1 分，非常消极。孩子经常皱眉，表现出沮丧或愤怒，烦躁，试图离开房间，或说不喜欢游戏。
2 分，比较消极。孩子表现出一些不高兴或沮丧的迹象，偶尔皱眉或大惊小怪，可能会简短地提到对游戏不感兴趣。
3 分，不消极也不积极。儿童参与游戏，但没有明显的高兴或不高兴的迹象。
4 分，比较积极。孩子偶尔微笑或大笑，在进行游戏时，会简单提及游戏中的乐趣。
5 分，非常积极。孩子精力充沛，经常笑，或说自己喜欢游戏。
二、讲完故事之后提出的问题

一级表征组

事实问题
1.在这个故事里面，狐狸用什么请仙鹤吃饭的？2.请仙鹤吃了什么？3.仙鹤吃饱了吗？4.为什么？
5.在这个故事里面，仙鹤用什么请狐狸吃饭的？6.请狐狸吃了什么？7.狐狸吃饱了吗？8.为什么？
心理状态问题
1.仙鹤在狐狸家吃完饭之前是高兴还是生气，为什么？2.狐狸在仙鹤家吃完饭之前是高兴还是生气？为什么？
3.仙鹤在狐狸家吃完饭之后是高兴还是生气，为什么？4.狐狸在仙鹤家吃完饭之后是高兴还是生气？为什么？

二级表征组

1.在这个故事里面，狐狸用什么请仙鹤吃饭的？2.请仙鹤吃了什么？3.仙鹤吃饱了吗？4.为什么？
5.在这个故事里面，仙鹤用什么请狐狸吃饭的？6.请狐狸吃了什么？7.狐狸吃饱了吗？8.为什么？
1.来狐狸家之前，狐狸说请仙鹤吃饭，仙鹤信了吗，为什么？2.来仙鹤家前，仙鹤说请狐狸吃饭，狐狸信了吗，为什么？
3.到狐狸家之后，狐狸说请仙鹤吃饭，仙鹤信了吗，为什么？4.到仙鹤家之后，仙鹤说请狐狸吃饭，狐狸信了吗，为什么？

English version of the experimental materials (translated from the Chinese version of the experimental materials)

Pretend Play Story Text: The Fox and the Crane's Invitation

Note: The black text represents the common parts of the story for both the secondary representation group and the primary representation group. The red text indicates content exclusive to the secondary representation group, and the blue text indicates content exclusive to the primary representation group.

Scene 1: In the Forest

Episode 1: The fox meets the crane and invites her to dinner

Narrator: A long time ago, in a forest, there lived a fox and a crane. One day, they met in the forest.

The fox thought: "Isn't that the crane? Hehehe, today I'm going to trick it; it won't expect this." (Today I'll invite it to dinner, and it will like it.)

The fox said, "Crane, are you hungry? Come to my house; I'll treat you to a meal!"

The crane thought: "How kind of the fox! It knows I'm a little hungry and has invited me to dinner." (I'm a bit hungry, so I'll go and eat.)

The crane replied, "Sure, sure!"

Scene 2: At the Fox's House

Episode 2: The crane visits the fox's home for dinner

Narrator: Then, the crane and the fox arrived at the fox's house.

[The fox cooked rice, placed it in a plate (bottle), and then placed the plate (bottle) on the table.]

The fox said, "Eat up, dear crane!"

[The crane used its long, thin beak to peck at the plate, tapping and tapping, but could not eat anything. (The crane stuck its long, thin beak into the bottle and ate quickly, finishing it all at once.)]

The fox thought: "Haha, I didn’t actually intend to treat it to dinner; it didn’t expect this." (The food I made is really delicious; it ate everything.)

The fox said, "Crane, was it delicious?"

Episode 3: The crane invites the fox to dinner in return

The crane thought: "I can't eat from a plate. The fox is using a plate to serve me; it must be doing this on purpose. To get back at it, I’ll play a trick on it too, and it won’t expect this." (The fox's food is really delicious, fragrant, and sweet. I really like it. The fox is so kind. To repay it, I’ll invite it to dinner, and it will really like it.)

The crane said, "It was delicious! Thank you so much today! To repay you, I’ll invite you to dinner!"

The fox thought: "I tricked it, and it still didn’t notice, haha, now it’s inviting me to dinner!" (I invited it, and now it’s inviting me to its house. Haha, this was a great invitation!)

The fox said, "Sure!"

Scene 3: At the Crane’s House

Episode 4: The fox goes to the crane’s home for dinner

Narrator: The next day, the fox arrived at the crane's house.

[The crane had already prepared the meal, pouring it into a bottle (plate) and then placing the bottle (plate) on the table.]

The crane said, "Dear fox, please eat!"

[The fox started to eat from the bottle, but it kept pressing its mouth against the bottle and sniffing it. No matter how hard it tried, the fox couldn't get anything from the bottle. (The fox started eating from the plate, eating the meat inside, drinking the fish soup. In no time, the fox had finished all the food on the plate.)]

The crane thought: "Haha, I didn't really mean to invite it to dinner; it didn't expect this." (The food I made is really delicious; it ate everything.)

The crane asked, "Fox, was it delicious?"

Episode 5: The fox leaves the crane's home

The fox thought: "The crane didn't really want to invite me to dinner! I should've known earlier!" (The food the crane served was really delicious! I'll definitely eat at its house again in the future!)

[The fox said nothing, its face flushed with embarrassment, and it walked away hungry. (The fox thanked the crane, patted its belly, and left to go home.)]

Records

1. Affective State of the Child During the Activity: ( )

Rating Scale:

1—Highly negative affect: Child frequently frowns, shows frustration or anger, fusses, attempts to leave the room, and/or verbally confirms dislike for task;

2—Somewhat negative affect: Child shows some signs of displeasure or frustration, occasionally frowns or fusses, may briefly mention disinterest in task;

3—Neutral affect: Child engages in task but shows no overt signs of enjoyment or displeasure;

4—Somewhat positive affect: Child occasionally smiles or laughs, is engaged in the task, may briefly mention enjoyment of task;

5—Highly positive affect: Child is energetic, frequently laughs, giggles, smiles, and/or verbally confirms enjoyment of task.

2. Questions After the Story

Primary Representation Group

Factual Questions

1. What did the fox use to invite the crane to dinner 2. What did the crane eat? 3. Was the crane full? 4. Why or why not?

5. What did the crane use to invite the fox to dinner? 6. What did the fox eat? 7. Was the fox full? 8. Why or why not?

Mental State Questions

1. Before the crane finished eating at the fox's house, was it happy or angry? Why? 2. Before the fox finished eating at the crane's house, was it happy or angry? Why?

3. After the crane finished eating at the fox's house, was it happy or angry? Why? 4. After the fox finished eating at the crane's house, was it happy or angry? Why?

Secondary Representation Group

Factual Questions

1. What did the fox use to invite the crane to dinner? 2. What did the crane eat? 3. Was the crane full? 4. Why or why not?

5. What did the crane use to invite the fox to dinner? 6. What did the fox eat? 7. Was the fox full? 8. Why or why not?

Mental State Questions

1. Before going to the fox’s house, when the fox said it would invite the crane to dinner, did the crane believe it? Why or why not? 2. Before going to the crane’s house, when the crane said it would invite the fox to dinner, did the fox believe it? Why or why not?

3. After arriving at the fox’s house, when the fox said it would invite the crane to dinner, did the crane believe it? Why or why not? 4. After arriving at the crane’s house, when the crane said it would invite the fox to dinner, did the fox believe it? Why or why not?
